# Conceptual Knowledge of Oral Health Among Primary School Teachers in Riyadh, Saudi Arabia—A Cross Sectional Survey

**DOI:** 10.3390/dj13010030

**Published:** 2025-01-14

**Authors:** Sanjeev. B. Khanagar, Rayan Albar, Abdullah Alghamdi, Sultan Alshamrani, Yousif Alhussain

**Affiliations:** 1 Preventive Dental Science Department, College of Dentistry, King Saud bin Abdulaziz University for Health Sciences, Riyadh 11426, Saudi Arabia; 2 King Abdullah International Medical Research Centre, Ministry of National Guard Health Affairs, Riyadh 11481, Saudi Arabia; 3 College of Dentistry, King Saud bin Abdulaziz University for Health Sciences, Riyadh 11426, Saudi Arabiaalghamdia067@ksau-hs.edu.sa (A.A.);

**Keywords:** oral health knowledge, oral health literacy, knowledge, conceptual, understanding, school teachers, educators

## Abstract

**Background/Objectives**: School teachers need to have a better understanding of oral health aspects as schools serve as an effective environment for learning. Educators hold a significant position in conveying the importance they attribute to oral health in their lives. According to the World Health Organization, school teachers should include oral health promotion activities to evaluate students’ oral health, track injuries, illnesses, and absenteeism related to oral health, advocate oral health prevention, and serve as role models. The aim of this study was to evaluate the conceptual understanding of oral health among primary school teachers in Riyadh, Saudi Arabia. **Methods**: Data for this study were collected from 404 primary school teachers using a structured and pre-validated comprehensive measure of oral health knowledge (CMOHK) questionnaire. **Results**: The distribution of CMOHK scores indicated that 247 (61.2%) participants demonstrated good conceptual knowledge, 95 (23.5%) participants exhibited fair knowledge, and 62 (15.3%) participants were classified as having poor knowledge. The male group exhibited lower values for the CMOHK score in comparison with the female group; however, these findings were not statistically significant. The findings indicated that the group of government school teachers exhibited higher values for the dependent variable CMOHK score compared to their private school counterparts. **Conclusions**: The average CMOHK score observed in this study is regarded as fair. It is crucial for school teachers to possess strong oral health knowledge, as they significantly influence the oral health knowledge and behaviors of children.

## 1. Introduction

Health literacy, a concept which first appeared in the 1970s, is fast changing from an individual to a public perspective, and one of its essential components is conceptual knowledge [1]. This pertains to the extent to which individuals are able to access, evaluate, and comprehend vital health information and services that are crucial for making informed decisions regarding their health [2].

Furthermore, understanding oral health literacy (OHL) is essential for identifying health-related information and applying it effectively to maintain a healthy lifestyle and attain oral health promotion objectives [3]. The importance of OHL promotion objectives has grown in recent years due to the shift in health services from a curative to preventive approach [4]. Research indicates that widespread limited literacy skills among adults significantly influence oral health disparities, thereby presenting a challenge to achieving enhanced oral health outcomes [5]. A number of studies indicate a correlation between inadequate oral health literacy and poor oral health outcomes, along with a lack of understanding of medical information and decreased dental appointments, which may hinder the attainment of optimal oral health [6,7]. Recent research has indicated that adolescents and college students in Saudi Arabia exhibit low levels of oral health literacy [8,9]. This deficiency in oral health knowledge was significantly associated with infrequent oral hygiene practices and irregular visits to dental facilities [8]. Oral health (OH) is an integral part of general health, well-being, and overall quality of life [10]. The World Health Organization (WHO) characterizes oral health as the condition of being devoid of oral and facial pain, oral and throat malignancies, oral infections, lesions, periodontal disease, dental caries, and edentulism [11]. Oral diseases constitute a public health issue owing to their widespread occurrence and considerable societal ramifications [12]. Despite a little enhancement in OH knowledge, the dental caries burden and a deficiency in OH awareness globally are still prevailing. The Global Burden of Disease Study 2019 estimated that oral disorders affect approximately 3.5 billion people worldwide [13]. Furthermore, research indicates that tooth decay affects 60–90% of school children and adults globally [11]. In Saudi Arabia, the prevalence of dental caries (DC) is reported to be high. A recent meta-analysis reported an average prevalence of 75.43% in deciduous dentition and 67.7% in permanent dentition [14]. In Medina City, Saudi Arabia, researchers reported that 87% of children with primary teeth had DC at the age of six, compared to 58% at the age of twelve and 83% with permanent teeth [15]. A study by Adam et al. [16] revealed that the prevalence of DC was 84% among children aged 5–7 years and 72% among those aged 12–15 years in Saudi Arabia. Alhabdan, Y. A. et al. [17] reported that the prevalence of DC was 83% among 6–8-year-old school children in Riyadh Saudi Arabia. This illustrates the increased prevalence of dental caries in children over the years and requires greater exploration and dissemination of knowledge, particularly as the majority of oral health disorders are preventable. Considering these findings, the present study focuses on primary school teachers because of their crucial role in the educational and teaching processes within society. Schools play a significant role in shaping habits and enhancing children’s understanding of OH as they spend a significant amount of time there. During the primary school period, children endure active development phases; hence, these phases are considered effective as children are more receptive and eager to learn, therefore representing an ideal time for establishing positive habits in their life [18]. Furthermore, teachers significantly contribute to the dissemination of knowledge and the cultivation of reflective future generations. Teachers have a pivotal role in enhancing students’ comprehension of oral health perceptions, which are essential for the development of good oral hygiene habits in early childhood [19]. According to the WHO, school teachers should include oral health promotion activities to evaluate students’ OH, track injuries, illnesses, and absenteeism related to OH, advocate OH prevention, and serve as role models [20]. Studies have also reported that teachers are as effective as dental professionals on delivering OH information to children [21]. Educators have the capacity to impart essential knowledge regarding OH care to children while also facilitating the early identification of oral diseases [22,23]. Research indicates that educators are capable of successfully providing OH education within school settings [24]. However, a lack of knowledge among teachers about OH may be a major barrier to the success of school efforts that promote health [25]. Almas K et al. [26] revealed that primary school teachers did not possess sufficient knowledge regarding oral health and that improving their knowledge is needed. Tikare et al. [27] indicated that the challenges in implementing health programs were primarily due to insufficient material resources, inadequate training, limited time, and a lack of administrative support, with the least significant factor being the attitude of teachers. Hence, it is essential for those in positions of authority and decision-making to enhance teachers’ competencies and motivate them to apply their knowledge to improve the health of children. Therefore, it is essential for teachers to undergo adequate training and participate in campaigns to enhance their role in promoting health within the school community. Researchers have conducted numerous studies to investigate the OH knowledge and attitudes of primary school teachers. The majority of this research indicates that most teachers in underdeveloped nations possess a limited understanding of oral health. Riyadh serves as the capital of Saudi Arabia and boasts the largest concentration of both public and private primary schools throughout the city. In 2021, the Riyadh region represented 22.8% of the total number of schools in the country [28]. Furthermore, the findings are pertinent to the wider population, considering the region’s degree of urbanization and it being culturally homogeneous [17]. Nevertheless, there is a lack of studies documenting the OH awareness of school teachers utilizing a standardized questionnaire, especially in Riyadh, Saudi Arabia. Therefore, the aim of this study was to evaluate the conceptual understanding of oral health among primary school teachers in Riyadh, Saudi Arabia.

## 2. Materials and Methods

### 2.1. Research Design

A cross-sectional analytical study was conducted after obtaining ethical clearance from the Institutional Review Board of the King Abdullah International Medical Research Center (KAIMRC) in Riyadh, Saudi Arabia (Reference Number: IRBC/1013/23; Study Number: SP23R/029/04, Approved on 7 May 2023).

### 2.2. Sample Size Estimation

The sample size was estimated based on the results presented in the literature [5,19]. The sample size was determined to ensure a power of 90% and a 95% confidence interval for a prevalence rate of 50% [29]. The required sample size for this study is 372 primary school teachers, encompassing both male and female participants.

### 2.3. Sampling Technique

A cluster sampling method was considered to recruit the participants necessary for this study. The city of Riyadh was divided into four regions: south, north, east, and west. A simple random sampling technique was adopted to select a minimum of 95 individuals from each region, dependent on meeting the eligibility criteria. Only participants who were present at the institutions on the day of sample selection were included in the sampling process, which could have resulted in a sampling error.

### 2.4. Eligibility Criteria

#### 2.4.1. Inclusion Criteria

Primary school teachers who provided a signed informed consent form and confirmed their willingness to participate were included.

#### 2.4.2. Exclusion Criteria

Primary school teachers unwilling to participate, administrative personnel, secondary school teachers, former primary school teachers, and those who have undergone prior training programs related to OH were excluded.

### 2.5. Data Collection

The data were obtained by utilizing a standardized pre-validated questionnaire which was distributed either in the form of personal printed copies or online via Google forms. The study participants were informed that no identifiable data would be collected, ensuring that the protection of their personal information and confidentiality would be completely upheld.

### 2.6. Data Collection Tool

A standardized pre-validated questionnaire comprising 23 questions was developed based on a review of similar research documented in the literature. The pre-validated questionnaire underwent translation from English to Arabic by a bilingual expert. Subsequently, it was translated back into English by an independent translator. A pilot study was conducted on 5% study samples from the same university’s primary school to evaluate the tool’s reliability. After two weeks, data were gathered from the same research participants in order to evaluate the test–retest reliability. An intra-class correlation coefficient value of 0.8 was obtained, suggesting that the instrument exhibited reliability. Therefore, the final version of the questionnaire did not undergo any additional modifications. When analyzing the final data, the answers from the pilot research were not included.

### 2.7. Data Analysis

The data collected were transferred into SPSS Statistical Software version 29 (IBM Corporation, Armonk, NY, USA). Data cleaning was conducted prior to the transfer to SPSS Statistical Software for analysis. Descriptive data were recorded, and a Chi-square test, an unpaired *t*-test, and ANOVA tests were performed to assess the significance of the findings. Further regression analysis was conducted to determine the odds associated with each significant finding. A *p* value < 0.05 was considered statistically significant.

## 3. Results

In the present study, 426 primary school teachers were approached to take part. Nonetheless, only 404 provided a response; therefore, the response rate stood at 96.6%.

### 3.1. Demographic Details of Study Participants

The demographic details revealed that 97 (24%) participants were from north Riyadh, 57 (14.10%) were from south Riyadh, 178 (44.10%) were from west Riyadh, and 72 (17.80%) were from east Riyadh. In the present study, 219 (54.20%) were male participants and 185 (45.80%) were female participants, while 183 (45.30%) were working in private schools and 221 (54.70%) were working in government schools (Table 1).

### 3.2. Details of the Comprehensive Measure of Oral Health Knowledge (CMOHK)

Details of the participants’ responses obtained using the comprehensive measure of oral health knowledge questionnaire are presented below. Overall, the percentage of correct responses was 66.21% (Table 2).

### 3.3. Comparison of Socio-Demographic Characteristics with CMOHK Score

The present evaluation utilized a one-way analysis of variance, revealing a significant difference between the categorical variable of school location (education office) and CMOHK score (F = 4.34, *p* = 0.005). The descriptive statistics indicated that the male group had lower CMOHK score values (M = 14.86, SD = 4.22) compared to the female group (M = 15.65, SD = 4.34). A *t*-test indicated that the difference in CMOHK scores between males and females was not statistically significant (*p* = 0.065, 95%). The descriptive statistics indicated that the government group exhibited higher values for the dependent variable CMOHK score (M = 15.37, SD = 3.96) compared to the private group (M = 15.05, SD = 4.66). Nonetheless, the *t*-test indicated that the difference between government and private regarding the dependent variable CMOHK score was not statistically significant, with *p* = 0.475 (Table 3).

### 3.4. Multivariate Logistic Regression Analysis of CMOHK Score

In the present study, scores ranging from 0 to 11 indicated “poor” conceptual oral health knowledge, scores from 12 to 14 indicated “fair” knowledge, and scores from 15 to 23 indicated “good” knowledge. The CMOHK scores indicated that 247 participants (61.2%) demonstrated good knowledge, 95 participants (23.5%) exhibited fair knowledge, and 62 participants (15.3%) were classified as having poor conceptual knowledge. A logistic regression analysis was conducted to assess the impact of school location, gender, and government /private schools on the variable CMOHK score to predict the value “Good”. The schools located in west Riyadh had an OR of 2.35 [CI 1.22–4.5] and were more likely to have good to fair knowledge compared to schools located in other regions in the city. Female participants had an OR of 1.73 [CI 1.15–2.6] and were more likely to have good to fair knowledge compared to male participants. Private school teachers had an OR of 2.35 [CI 1.22–4.5] and were more likely to have good to fair knowledge compared to public school teachers (Table 4).

## 4. Discussion

Educators play pivotal roles in the lives of young learners, significantly impacting not only the impartation of academic knowledge but also the shaping of health-related behaviors. This study emphasizes that early childhood is a critical phase for establishing oral health habits, positioning teachers as essential in advocating preventive measures. Children face an increased likelihood of dental caries if their caregivers fail to provide consistent preventative care guidance or lack understanding. This emphasizes caregivers and school teachers as more appropriate candidates for OH education initiatives than parents [30]. Due to their expertise and educational qualifications, teachers serve as a dependable source for imparting knowledge. Consequently, educators must possess a favorable disposition and knowledge of oral health to cultivate effective preventative oral behaviors [25]. Bhadauria, U.S et al. [31] reported that school teachers can enhance the oral health knowledge and behaviors of children through counseling and reinforcement. According to Aldowah et al. [32], teachers with advanced degrees and with substantial work experience had a better understanding of dental caries because of the knowledge they had gained over their teaching careers. Rajab et al. [33] reported a link between parents with higher levels of education and a greater concern for their child’s dental health. The data regarding OH awareness among primary school teachers in Saudi Arabia sre insufficient, with most research exhibiting issues related to gender bias, sample size, or the application of standardized assessment instruments. Prior research publications have focused on OH literacy [5]. This study assessed the conceptual knowledge of oral health among primary school teachers in Riyadh, Saudi Arabia.

The current study revealed that a significant percentage of participants exhibited strong conceptual knowledge (61.2%), while a notable proportion were classified as having fair (23.5%) or poor (15.3%) knowledge levels. The findings are in accordance with a study reported by Jagan et al. [5] in South India utilizing the same tool; however, their research indicated that only 44% of teachers exhibited a strong understanding of conceptual oral health knowledge. The observed changes may result from heightened awareness among school teachers facilitated by social media, health messages, and oral health campaigns. This distribution highlights the need for focused interventions to enhance overall OH. Aldowah et al. [32] reported additional factors that may impact oral health knowledge among school teachers. They indicated that the age and education level of school teachers were significantly linked to their OH literacy. Teachers in younger age groups demonstrated greater knowledge than their older counterparts, while those holding a bachelor’s degree in education exhibited higher odds compared to individuals with other educational qualifications. Previous studies conducted in Saudi Arabia have yielded satisfactory results in terms of OH knowledge despite the use of various questionnaires and assessment tools [25,27,32,34,35,36]. On the contrary, few studies concluded that OH knowledge was limited among school teachers and that further improvement is needed [12,15,26].

In the present study, demographic variables, especially gender and the type of school, showed differences in terms of CMOHK score; however, these differences were not statistically significant. The findings of this study indicate that being female enhances the probability of having a “good” CMOHK score. This finding is consistent with numerous other studies that emphasize the gender disparities in oral health knowledge among school teachers. All of these studies indicate that females tend to have greater oral health knowledge compared to males [5,27,32,34,35,36]. This could be attributed to the growing presence of female educators in schools, along with the elevated oral hygiene consciousness among female teachers [32]. It has been noted that a significant number of these studies included a greater proportion of female participants in comparison to their male counterparts in the teaching profession [5,32,36]. However, a study conducted by Almas K et al. [26] found no difference in knowledge between male and female teachers.

In this study, private school teachers exhibited a higher probability of achieving a “good” CMOHK score in comparison with their public school counterparts; however, these findings were not statistically significant. These findings could be attributed to the affordability and utilization of preventive oral health care among private school teachers, as reported in previous studies in Saudi Arabia [37]. Similar research conducted in Nepal indicated that private school teachers possessed superior OH knowledge in comparison to public school teachers [38]. A study in the Emirate of Sharjah assessing OH knowledge among daycare caretakers revealed that those in public centers possessed a greater degree of knowledge than their counterparts in private centers [30]. However, Tikare et al. [27] concluded that there is no difference in knowledge scores between government and private primary school teachers.

Educational initiatives and programs in educational institutions can effectively improve the oral health awareness of both educators and students. These efforts empower teachers by providing them with correct information and excellent teaching methods to help children adopt improved oral hygiene practices. Priya, H et al. [22] assessed the effectiveness of an OH training program for school teachers in India, indicating that most teachers recognized the necessity of promotional training and exhibited a substantial increase in mean knowledge scores following a 1-day training session. Alshemari et al. [39] indicated in their survey that 74% of teachers expressed a willingness to engage in online courses aimed at promoting oral health among students in primary schools. Aldowah et al. [32] reported in their survey that 96.8% of participants believe that dental health education needs to be incorporated into the primary school curriculum and that teachers should undergo regular training in dental health education. Aljanakh et al. [34] reported that 96% of teachers express interest in serving as oral health promoters, while 84% believe that teachers should receive training in OH education.

This study addresses the limitation of utilizing a standardized questionnaire for evaluating OH knowledge by using the CMOHK questionaire, a standardized instrument for assessing conceptual understanding. A study conducted by Macek et al. [40] comparing three oral health literacy instruments revealed that CMOHK scores displayed greater variability in assessing patients’ oral health than REAL-M and TOFHLA. Therefore, the results of this study may be utilized to enhance oral health understanding among school educators. Vozza et al. [41] discovered that school preventive initiatives are more beneficial for children’s learning, particularly when knowledge acquisition is accompanied by the application and validation of theoretical and practical skills related to OH. Saccomanno et al. [18] discovered that children were willing to acquire knowledge in an academic setting, and that educational programs constituted a successful strategy. Additionally, it is recommended that nationwide studies be conducted to examine the knowledge of school teachers and students, with the aim of evaluating and comparing results while resolving the obstacles encountered by teachers., thus contributing to the development of an informed generation with enhanced oral health and knowledge and ultimately lowering the burden of oral disease in the future. A few of the limitations of this study were that during the data collection phase, the data were collected from participants who were present at the institutions on the day of sample selection, which could have resulted in a sampling error and could have resulted in the over-representation of certain types of teachers. The present study was only limited to Riyadh, and hence future research should consider different regions of the country. In the present study, it was noted that there was no uniform distribution of the study participants representing the regions of Riyadh city. The majority of the responses were from the northern part of Riyadh and the least were from the southern part of Riyadh. These variations could be due to the time the teachers were approached in the schools. Teachers already have many tasks during their school working hours, and reaching them in their free time was a little challenging during the data collection process. Future studies should also consider assessing the relationship of the teachers’ years of experiences with their CMOHK scores.

Recommendations: The implications of these findings reveal the need to conduct future research in different regions of the country. These findings should be considered when developing oral health educational programs tailored to school teachers. These effective oral health programs can empower teachers by providing them with correct information and help them in adopting excellent teaching methods to help children develop better oral health knowledge and behaviors. The results of our study emphasize the necessity of developing and organizing oral health educational initiatives for educators. Disseminating oral health knowledge through targeted educational programs and incorporating oral health education into the school curriculum can improve teachers’ conceptual understanding of oral health, thereby enhancing their capacity to serve as providers of oral health education within the school environment. Future studies should also consider the role of parents in imparting oral health behaviors and practices among their children.

## 5. Conclusions

It is crucial for school teachers to have a better conceptual understanding of OH aspects as schools serve as an effective environment for learning. Educators play a significant role in conveying the importance they attribute to OH in their lives. Effective tailored oral health programs designed for school teachers can empower them by providing them with correct information and help them in adopting excellent teaching methods to help children develop better oral health knowledge and behaviors. In the present study, the overall distribution of CMOHK scores revealed that 247 (61.2%) participants had good, 95 (23.5%) had fair, and 62 (15.3%) had poor conceptual knowledge. The male group exhibited lower CMOHK scores compared to the female group; however, these findings were not statistically significant. The findings indicate that the group of government school teachers exhibited higher values for the dependent variable CMOHK score compared to their private school counterparts. In conclusion, the average CMOHK score observed in this study is regarded as fair.

## Figures and Tables

**Table 1:** Demographic details of study participants.

Statements	Responses	N	%
Teachers willingness to participate	Agree to participate	404	94.60%
	Disagree to participate	22	5.40%
School (education office) location	North of Riyadh	97	24.00%
	South of Riyadh	57	14.10%
	East of Riyadh	178	44.10%
	West of Riyadh	72	17.80%
Gender	Male	219	54.20%
	Female	185	45.80%
Type of school	Private School	183	45.30%
	Government School	221	54.70%
Total		404	100%

**Table 2:** Details of the comprehensive measure of oral health knowledge (CMOHK) questionnaire.

Statements	Responses	N	%	Correct Responses
Q1	Gingiva	67	16.60%	269 (66.60%)
	Canine	4	1.00%	
	Palate	269	66.60%	
	Gland	17	4.20%	
	Don’t know	47	11.60%	
Q2	Incisor	1	0.20%	268 (66.30%)
	Tonsils	116	28.70%	
	Sinus	3	0.70%	
	Uvula	268	66.30%	
	Don’t Know	16	4.00%	
Q3	10	98	24.30%	198 (49.00%)
	20	198	49.00%	
	32	38	9.40%	
	45	0	0.00%	
	Dont Know	70	17.30%	
Q4	10	10	2.50%	335 (82.90%)
	20	25	6.20%	
	32	335	82.90%	
	45	9	2.20%	
	Don’t Know	25	6.20%	
Q5	About 1 year old	150	37.10%	176 (43.60%)
	About 3 year old	35	8.70%	
	About 6 year old	176	43.60%	
	About 13 year old	25	6.20%	
	Don’t Know	18	4.50%	
Q6	Replacing missing teeth	10	2.50%	373 (92.30%)
	Preventing tooth decay	10	2.50%	
	Making teeth whiter	5	1.20%	
	Straightening crooked teeth	373	92.30%	
	Don’t know	6	1.50%	
Q7	It kills germs in water	58	14.40%	255 (63.10%)
	It makes the water tastes better	10	2.50%	
	It protects teeth from tooth decay	255	63.10%	
	It protects teeth from gum diseases	37	9.20%	
	Don’t know	44	10.90%	
Q8	Replacing missing teeth	364	90.10%	364 (90.10%)
	Preventing tooth decay	12	3.00%	
	Making teeth whiter	7	1.70%	
	Straightening crooked teeth	9	2.20%	
	Don’t know	12	3.00%	
Q9	Incisor	32	7.90%	189 (46.80%)
	Dentine	148	36.60%	
	Premolar	0	0.00%	
	Enamel	189	46.80%	
	Don’t know	35	8.70%	
Q10	Every month	34	8.40%	300 (74.30%)
	Two times per year	300	74.30%	
	One time per year	36	8.90%	
	When they have tooth ache	19	4.70%	
	Don’t know	15	3.70%	
Q11	Salt	7	1.70%	369 (91.30%)
	Spices	7	1.70%	
	Fat	7	1.70%	
	Sugar	369	91.30%	
	Don’t know	14	3.50%	
Q12	The childs teeth might not come in the right time	39	9.70%	192 (47.50%)
	The child might get gum disease	33	8.20%	
	The child might get tooth decay	192	47.50%	
	The child might get Crooked tooth	49	12.10%	
	Don’t know	91	22.50%	
Q13	Using tooth pick after every meal	10	2.50%	348 (86.10%)
	Drinking sugar free soda	11	2.70%	
	Rinsing with mouthwash like Listerine	21	5.20%	
	Brushing and flossing everyday	348	86.10%	
	Don’t know	14	3.50%	
Q14	Prescribing antibiotics	10	2.50%	348 (86.10%)
	Placing filling in the mouth	348	86.10%	
	Pulling the tooth	8	2.00%	
	Adding dental implant	10	2.50%	
	Dont know	28	6.90%	
Q15	Removing the tooth enamel	16	4.00%	278 (68.80%)
	Removing the tooth dentine	23	5.70%	
	Removing the tooth nerve	278	68.80%	
	Removing the tooth cusp	17	4.20%	
	Don’t know	70	17.30%	
Q16	Gum disease	19	4.70%	318 (78.70%)
	Tooth decay	318	78.70%	
	Cold sores	5	1.20%	
	Mouth cancer	17	4.20%	
	Don’t know	45	11.10%	
Q17	Gingivitis	256	63.40%	256 (63.40%)
	Periodontitis	93	23.00%	
	Canker sores	11	2.70%	
	Leukoplakia	6	1.50%	
	Don’t know	38	9.40%	
Q18	Biting your finger nails	39	9.70%	216 (53.90%)
	Eating spicy foods	26	6.50%	
	Drinking too much coffee	31	7.70%	
	Smoking cigarettes	216	53.90%	
	Don’t know	89	22.20%	
Q17	Eating foods like apples	12	3.00%	311 (77.00%)
	Rinsing with mouthwashes like Listerine	24	5.90%	
	Brushing and flossing	35	8.70%	
	Getting dental cleaning	311	77.00%	
	Don’t know	22	5.40%	
Q20	Fluorosis	16	4.00%	266 (65.80%)
	Periodontal disease	266	65.80%	
	Halitosis	35	8.70%	
	Mouth cancer	10	2.50%	
	Don’t know	77	19.10%	
Q21	High cholesterol	33	8.20%	243 (60.10%)
	Hepatitis	19	4.70%	
	High blood pressure	11	2.70%	
	Diabetes	243	60.10%	
	Don’t know	98	24.30%	
Q22	A sore that lasts more than 2 weeks	149	36.90%	149 (36.90%)
	Pain when you open mouth	25	6.20%	
	Gums that bleeds when you brush	20	5.00%	
	Teeth that have black spot on them	34	8.40%	
	Don’t know	176	43.60%	
Q23	Men younger than 40 years of age	12	3.00%	130 (32.20%)
	Women younger than 40 years of age	17	4.20%	
	Men older than 40 years of age	130	32.20%	
	Women older than 40 years of age	38	9.40%	
	Don’t know	207	51.20%	
Overall % of Correct Responses				66.21%

**Table 3:** Comparison of socio-demographic characteristics with CMOHK score.

Parameters	Responses	N	Mean Score	SD	*p* Value
School location	South Riyadh	57	16.33	4.36	0.005 *
	East Riyadh	178	14.89	3.78	
	West Riyadh	72	16.28	5.52	
	North Riyadh	97	14.41	3.86	
Gender	Male	219	14.86	4.22	0.065
	Female	185	15.65	4.34	
Type of school	Government	221	15.37	3.96	0.475
	Private	183	15.05	4.66	

* = statistically significant.

**Table 4:** Multivariate logistic regression analysis of CMOHK score.

Parameters		CMOHK Score				
		Good	Fair		Poor	
		N	N	Beta (OR (95% CI))	N	Beta (OR (95% CI))
School location (education office)	North Riyadh	51	27	Reference Group	19	Reference Group
	South Riyadh	39	11	0.67 (1.95 (0.98–3.88))	7	−0.48 (0.62 (0.28–1.37))
	East Riyadh	105	50	0.26 (1.3(0.79–2.13))	23	0.01 (1.01 (0.58–1.76))
	West Riyadh	52	7	0.85 (2.35 (1.22–4.5)) *	13	−1.28 (0.28 (0.11–0.68))
Gender	Male	121	63	Reference Group	35	Reference Group
	Female	126	32	0.55 (1.73 (1.15–2.6)) *	27	−0.66 (0.52 (0.32–0.84)) *
Type of school	Government	137	57	Reference Group	27	Reference Group
	Private	110	38	0.85 (2.35 (1.22–4.5))	35	−0.28 (0.75 (0.47–1.2))

* = statistically significant.

## Data Availability

The data are contained within the article.

## References

[1] Sorensen K., van den Broucke S., Fullam J., Doyle G., Pelikan J., Slonska Z., Brand H. (2012). Health Literacy and Public Health: A Systematic Review and Integration of Definitions and Models. BMC Public Health.

[2] ODPHP Health History of Health Literacy Definitions—Healthy People 2030. https://odphp.health.gov/healthypeople/priority-areas/health-literacy-healthy-people-2030/history-health-literacy-definitions.

[3] Batista M.J., Lawrence H.P., de Sousa M.d.L.R. (2017). Oral Health Literacy and Oral Health Outcomes in an Adult Population in Brazil. BMC Public Health.

[4] Ahmed W., Shah S.M.A., Khayyam U., Sheikh T., Anwer N. (2018). Measuring Oral Health Literacy in Dental Patients: Contribution towards Preventive Dentistry in Pakistan. J. Pak. Dent. Assoc..

[5] Jagan P., Fareed N., Battur H., Khanagar S., Manohar B. (2018). Conceptual Knowledge of Oral Health among School Teachers in South India, India. Eur. J. Dent..

[6] Kanupuru K.K., Fareed N., Sudhir K. (2015). Relationship between Oral Health Literacy and Oral Health Status among College Students. Oral Health Prev. Dent..

[7] Lapidos A., Shaefer H.L., Gwozdek A. (2015). Toward a Better Understanding of Dental Appointment-Keeping Behavior. Community Dent. Oral Epidemiol..

[8] Alzeer M., AlJameel A., Rosing K., Ozhayat E. (2024). The Association between Oral Health Literacy and Oral Health-Related Behaviours among Female Adolescents in the Kingdom of Saudi Arabia: A Cross-Sectional Study. Saudi Dent. J..

[9] Kandasamy G., Almaghaslah D., Vasudevan R., Shorog E., Alshahrani A.M., Alsawaq E.M., Alzlaiq W.A., Prabahar K., Veeramani V.P., Alshareef H. (2023). Assessment of Oral Health Literacy and Oral Health-Related Quality of Life in Saudi University Students: A Cross-Sectional Study. J. Oral Rehabil..

[10] Baiju R. (2017). Oral Health and Quality of Life: Current Concepts. J. Clin. Diagn. Res..

[11] Petersen P.E. (2003). The World Oral Health Report 2003: Continuous Improvement of Oral Health in the 21st Century—The Approach of the WHO Global Oral Health Programme. Community Dent. Oral Epidemiol..

[12] Khan N., Al Zarea B., Al Mansour M. (2001). Dental Caries, Hygiene, Fluorosis and Oral Health Knowledge of Primary School Teachers of Riyadh, Saudi Arabia. Saudi Dent. J..

[13] World Health Organization (2022). Global Oral Health Status Report.

[14] Khan S.Q., Alzayer H.A., Alameer S.T., Khan M.A., Khan N., AlQuorain H., Gad M.M. (2024). SEQUEL: Prevalence of Dental Caries in the Saudi Arabia: A Systematic Review and Meta-Analysis. Saudi Dent. J..

[15] Al-Tamimi S., Petersen P.E. (1998). Oral Health Situation of Schoolchildren, Mothers and Schoolteachers in Saudi Arabia. Int. Dent. J..

[16] Adam T.R., Al-Sharif A.I., Tonouhewa A., AlKheraif A.A. (2022). Prevalence of Caries among School Children in Saudi Arabia: A Meta-Analysis. Adv. Prev. Med..

[17] Alhabdan Y.A., Albeshr A.G., Yenugadhati N., Jradi H. (2018). Prevalence of Dental Caries and Associated Factors among Primary School Children: A Population-Based Cross-Sectional Study in Riyadh, Saudi Arabia. Environ. Health Prev. Med..

[18] Saccomanno S., de Luca M., Saran S., Petricca M.T., Caramaschi E., Mastrapasqua R.F., Messina G., Gallusi G. (2023). The Importance of Promoting Oral Health in Schools: A Pilot Study. Eur. J. Transl. Myol..

[19] Patil D., Khakhar P., Katge F., Bhanushali N., Bhanushali P., Deshpande S. (2022). Knowledge, Attitude, and Practice of School Teachers Regarding Dental Caries for School Children in Navi Mumbai, India. Indian J. Dent. Sci..

[20] World Health Organization (2003). Oral Health Promotion: An Essential Element of a Health-Promoting School. https://iris.who.int/handle/10665/70207.

[21] Oyapero A., Edomwonyi A., Adeniyi A., Adedigba M. (2020). Use of Teachers as Agents of Oral Health Education: Intervention Study among Public Secondary School Pupils in Lagos. J. Fam. Med. Prim. Care.

[22] Priya H., Khurana C., Kharbanda O., Bhadauria U., Das D., Ravi P., Dev D.M. (2020). Effectiveness of an Oral Health Training Program for School Teachers in India: An Interventional Study. J. Educ. Health Promot..

[23] Gargano L., Mason M.K., Northridge M.E. (2019). Advancing Oral Health Equity through School-Based Oral Health Programs: An Ecological Model and Review. Front. Public Health.

[24] Eley C., Weston-Price S., Young V., Hoekstra B., Gadhia T., Muirhead V., Robinson L., Pine C., McNulty C. (2019). Using Oral Hygiene Education in Schools to Tackle Child Tooth Decay: A Mixed Methods Study with Children and Teachers in England. J. Biol. Educ..

[25] Wyne A.H., Al-Ghorabi B.M., Al-Asiri Y.A., Khan N.B. (2002). Caries Prevalence in Saudi Primary Schoolchildren of Riyadh and Their Teachers’ Oral Health Knowledge, Attitude and Practices. Saudi Med. J..

[26] Almas K., Al-Malik T.M., Al-Shehri M.A., Skaug N. (2003). The Knowledge and Practices of Oral Hygiene Methods and Attendance Pattern among School Teachers in Riyadh, Saudi Arabia. Saudi Med. J..

[27] Tikare S., AlQahtani N. (2017). Oral Health Knowledge and Attitudes of Primary School Teachers toward School-Based Oral Health Programs in Abha-Khamis, Saudi Arabia. Saudi J. Oral Sci..

[28] Jamal S. Saudi Arabia Education Report 2021 Opportunities in the Sector. https://www.knightfrank.com.sa/en/research/saudi-arabia-education-report-2021-7999.aspx.

[29] Ngamjarus C. (2016). N4Studies: Sample Size Calculation for an Epidemiological Study on a Smart Device. Siriraj Med. J..

[30] El Batawi H.Y., Fakhruddin K.S. (2017). Impact of Preventive Care Orientation on Caries Status among Preschool Children. Eur. J. Dent..

[31] Bhadauria U.S., Mathur R.V., Agarwal A., Shukla R., Godha S., Maheshwari R. (2020). Impact of Counseling and Reinforcement by School Teachers on Behavior Change in Children: A One -Year Follow-up Study. J. Educ. Health Promot..

[32] Aldowah O., Assiry A.A., Mujallid N.F., Ashi F.N., Abduljawad F., Al-Zahrani M.M., Ezzaddin R., Karobari M.I. (2023). Assessment of Oral Health Knowledge, Literacy, and Attitude among Schoolteachers towards Oral Health—A Cross-Sectional Study. BMC Oral Health.

[33] Rajab L.D., Petersen P.E., Bakaeen G., Hamdan M.A. (2002). Oral Health Behaviour of Schoolchildren and Parents in Jordan. Int. J. Paediatr. Dent..

[34] Aljanakh M., Siddiqui A.A., Mirza A.J. (2016). Teachers’ Knowledge about Oral Health and Their Interest in Oral Health Education in Hail, Saudi Arabia. Int. J. Health Sci..

[35] Ahmad M.S. (2015). Oral Health Knowledge and Attitude among Primary School Teachers of Madinah, Saudi Arabia. J. Contemp. Dent. Pract..

[36] Shaheen R., AlShulayyil M., Baseer M.A., Bahamid A.A.S., AlSaffan A.D., Al Herbisch R. (2021). Self-Reported Basic Oral Health Knowledge of Primary School Students and Teachers in Rural Areas of Saudi Arabia. Clin. Cosmet. Investig. Dent..

[37] Albisher G.M., Alghamdi H.M., AlAbbadi S.H., Almukhyzim N.I., Fayez R.A.A., Alamrani H.A., Saffan A.D.A. (2021). Oral Health Knowledge among Private Primary School Teachers in Riyadh City, Kingdom of Saudi Arabia. Arch. Pharm. Pract..

[38] Singh H., Chaudhary S., Gupta A., Bhatta A. (2021). Oral Health Knowledge, Attitude, and Practices among School Teachers in Chitwan District, Nepal. Int. J. Dent..

[39] Alshemari M.A., Alkandari S.A. (2021). Oral Health Knowledge and Attitudes towards Oral Health Education among Elementary School Teachers in Kuwait. Oral Health Prev. Dent..

[40] Macek M.D., Haynes D., Wells W., Bauer-Leffler S., Cotten P.A., Parker R.M. (2010). Measuring Conceptual Health Knowledge in the Context of Oral Health Literacy: Preliminary Results. J. Public Health Dent..

[41] Vozza I., Capasso F., Calcagnile F., Anelli A., Corridore D., Ferrara C., Ottolenghi L. (2019). School-Age Dental Screening: Oral Health and Eating Habits. Clin. Ter..

